# Renal-Type Clear Cell Carcinoma of the Prostate: A Histopathological Case Report of a Rare and Underrecognized Variant

**DOI:** 10.3390/reports9010063

**Published:** 2026-02-14

**Authors:** George Stoyanov, Dobri Marchev, Pavel Pavlov, Hristo Popov

**Affiliations:** 1 Department of Pathology, Multiptofile Hospital for Active Treatment, 9700 Shument, Bulgaria; 2 Department of Urology, Multiptofile Hospital for Active Treatment, 9700 Shument, Bulgaria; dobrimarchev@abv.bg; 3 Department of Pathology, Complex Oncology Center, 9700 Shumen, Bulgaria; pavel.slavov.pavlov@gmail.com; 4 Department of General and Clinical Pathology, Forensic Medicine and Deontology, Medical University—Varna, 9000 Varna, Bulgaria; popov12@abv.bg

**Keywords:** prostate, malignancy, acinar adenocarcinoma, clear cells, renal-type clear cell acinar adenocarcinoma

## Abstract

**Background and Clinical Significance**: Prostatic malignancies are amongst the leading malignancies in incidence. They represent a mixed group of conditions, predominantly characterized by adenocarcinomas, which are themselves predominantly acinar. **Case Presentation**: Herein, we present a morphological case report of a 73-year-old male who underwent transrectal ultrasound-guided needle biopsy due to elevated PSA levels (18.61 ng/mL). Histopathology of the biopsy specimen was represented by pleomorphic cells with predominantly clear cytoplasm, with relatively eccentric, pyknotically appearing nuclei with hyperchromatic chromatin and no visible nucleoli. After imaging excluded concomitant renal malignancy and confirmatory immunohistochemistry was carried out, the patient was diagnosed with renal-type clear cell carcinoma of the prostate. **Conclusions**: While unrecognized by the WHO due to its rarity, renal-type clear cell acinar adenocarcinoma of the prostate is a unique type of prostatic malignancy, which, due to its morphological appearance, necessitated careful differential diagnosis.

## 1. Introduction and Clinical Significance

Despite developing only in males, prostate carcinoma is the fourth most commonly diagnosed malignancy overall and, depending on the region, either the first or second most common among males as per the latest GLOBOCAN data [1,2]. Despite its high incidence, however, the malignancy-related death rate of prostatic carcinoma are significantly less than those of other malignancies diagnosed less often, such as liver, colorectal, gastric, pancreatic and esophageal malignancies [1]. This can be attributed both to the historical indolent behavior of most malignancies of the prostate and the implementation of screening methods such as PSA level monitoring in males and MRI, aiding in the early diagnosis and hence treatment of non-advanced cases.

As a gland, most of the malignancies that develop within the prostate are adenocarcinomas, although exotic entries of non-glandular origin also exist, such as neuroendocrine and mesenchymal ones [3]. The WHO classification of urinary and male genital tumors currently recognizes two main types of adenocarcinomas of the prostate—acinar and ductal [3]. Acinar adenocarcinomas develop from the acini of the gland. They are represented by a number of subtypes and patterns other than the conventional prostatic adenocarcinoma, including atrophic pattern adenocarcinoma, pseudohyperplastic, microcystic, foamy gland, mucinous, signet-ring, sarcomatoid, pleomorphic giant cell, and PIN-like adenocarcinomas [3]. Ductal adenocarcinomas are extremely rare, with their pure form accounting for less than 0.5% of all cases and as a component of a mixed ductal-acinar malignancy in less than 3% of all cases [4,5,6].

The etiology of ductal carcinomas is also widely disputed, as for a long time, they were thought to derive from Müllerian remnants within the gland; however, since the recognition of identical immunohistochemical and molecular profiles of both ductal and acinar adenocarcinomas as well as evidence of clonal connection in mixed cases, nowadays, ductal prostatic adenocarcinomas are thought more of as a special type with divergent differentiation [6,7,8].

Despite the frequency of prostatic adenocarcinomas and the detailed nature of their histological classification, there still remain some morphologically distinct but unrecognized types of prostatic adenocarcinoma.

## 2. Case Presentation

A 73-year-old Caucasian male patient with a previous medical history of long-lasting hypertension and cerebrovascular incident three years prior was referred from outpatient urology for prostate biopsy due to significantly elevated PSA levels of 18.61 ng/mL. The patient reported no family history of malignancy, and the physical exam was unremarkable, apart from a firm enlarged prostate on digital rectal exam.

Transrectal ultrasound-guided needle biopsy of the prostate was performed under general anesthesia and went uncomplicated with an uneventful postoperative period.

**Figure 1:**
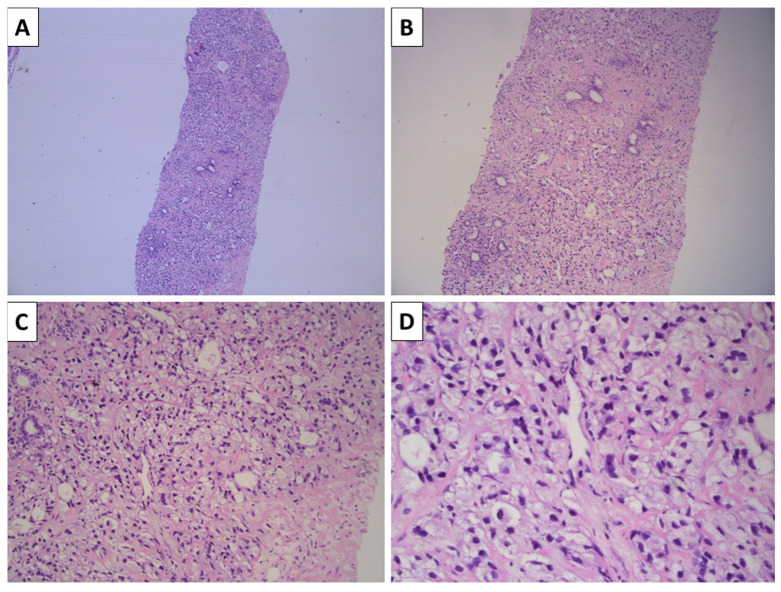
Histopathology of the lesion. (**A**): Prostatic core specimen involved with a solid tumor process—H&E stain, original magnification 40×; (**B**): solid tumor intersecting the stroma and acini of the prostate—H&E stain, original magnification 100×; (**C**): solid tumor comprising pale cells—H&E stain, original magnification 200×; (**D**): clear cells with relatively eccentric nuclei with uneven border and hyperchromatic nuclei, some with pyknotic features and no visible nucleoli—H&E stain, original magnification 400×.

The specimen sent for histology showed prostatic parenchyma involved with a tumor process growing in the form of solid nests represented by large cells differing in size with predominantly clear cytoplasm with relatively eccentric, pyknotically appearing nuclei with hyperchromatic chromatin and no visible nucleoli (Figure 1). The total area of the tumor involved 40% of the cores sent for histopathology.

**Figure 2:**
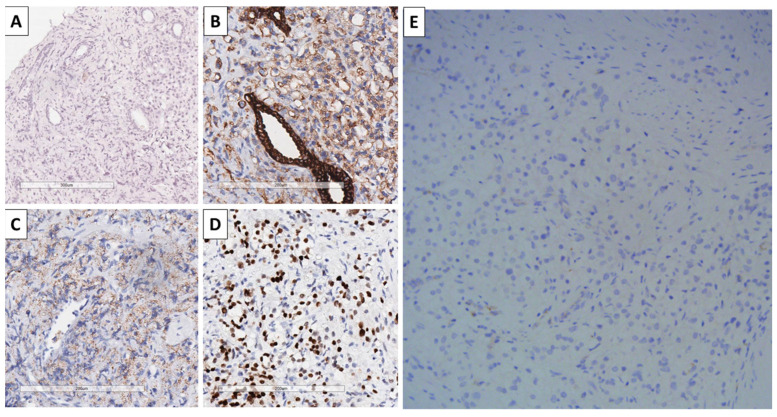
Immunohistochemistry of the tumor. (**A**): CD68, negative reaction, original magnification 100×; (**B**): CK AE1/AE3, positive internal control, and positive reaction within the tumor cells, original magnification 200×; (**C**): AMACR, positive reaction, original magnification 200×; (**D**): NKX3.1, positive reaction, original magnification 200×; (**E**): PAX8, negative reaction, original magnification 200×.

Due to the unconventional morphology of the tumor, immunohistochemistry was performed, and imaging was suggested to the clinician with the goal of differentiating from clear cell renal carcinoma metastatic to the prostate. Neither renal ultrasound nor CT showed evidence of a tumor process involving either of the kidneys. Immunohistochemistry of the tumor was negative for CD68 and positive for CK AE1/AE3, AMACR, and NKX3.1, while being negative for PAX8 (Figure 2). Hence, the diagnosis of renal-type clear cell carcinoma of the prostate was established and graded as Gleason 5 + 5 = 10, grade group 5, and the patient was referred to the oncological committee for treatment and follow-up.

Five months after the biopsy, the patient underwent treatment with androgen blockade and local radiotherapy and is currently being followed-up with at another institution, without disease progression being noted.

## 3. Discussion

As already mentioned, renal-type clear cell carcinomas of the prostate are rare and currently underrecognized malignancies of the prostate. There have only been a handful of case reports on these rare tumors [9,10,11,12,13,14,15].

The rarity of the condition requires a careful approach for its proper diagnosis. Chief among these is the differentiation from the much more common malignancy with identical morphology—clear cell renal cell carcinoma, which is the dominant form of renal malignancy [1]. Although rare, clear cell renal cell carcinomas can metastasize below the level of development of the primary tumor through both canalicular spread through ureters and hematogenous pathways, particularly with venous outflow obstruction of the kidney [16]. Thus, imaging of the kidneys is the first crucial step in differentiating these malignancies.

Secondly, as other malignancies can have a similar morphological appearance to clear cell renal cell carcinoma, immunohistochemistry is invaluable for diagnosis. First, clear cell renal cell carcinoma must be distinguished from benign conditions, such as xanthogranulomatous prostatitis, which is characterized by extensive accumulation of foamy macrophages in the prostate parenchyma and can be identified using CD68, a sophisticated macrophage marker. Secondly, it must be differentiated from other malignancies, such as clear cell sarcomas and clear cell melanoma, which are negative for CK AE1/AE3. Thirdly, while renal cell carcinoma is typically positive for CD10, PAX8, and RCC, as shown in the published cases of clear cell renal type carcinomas, some individual cases test negative for these markers or show weak CD10 positivity [10,11,12,13,14,15]. Another marker reported as negative is CK7, which further aids in differentiating from other similar malignancies in terms of morphology, including some hepatocellular carcinomas and gastric and pancreatic carcinomas, while being consistently negative in prostatic ones [12,13,14]. From cases that report AMACR testing, as in our case, it has been unanimously reported as positive in all cases, unlike PSA, for which some of the reported cases are negative [13,14]. NKX3.1 is a reliable prostate marker; however, this report seems to be the first one to depict it as positive in these malignancies, as in previously published cases, it had not been reported. A summary of immunohistochemical marker is presented in Table 1.

The exact placement of renal-type clear cell carcinomas of the prostate in the WHO classification as either ductal or acinar could be widely debated upon if the entry is recognized. While the cell morphology and rarity of the condition would better fit in the ductal category, as originating from Müllerian remnants, the specific immunoprofile reported, which is PAX8 negative, AMACR, and often PSA positive, identifies a clear cell renal-type prostatic adenocarcinoma as a rare entry in the acinar category, with possible divergent differentiation [10,11,12,13,14,17,18,19]. PAX8 in this regard is a valuable and sophisticated marker, as tumors with this location and similar morphology, positive for PAX8, should be regarded as renal cell malignancies, either metastatic or developing of renal rest within the prostate.

The rarity of reported cases may also not reflect the true incidence of the condition, as it is often misinterpreted as other WHO-recognized types of acinar adenocarcinoma of the prostate, such as signet ring or foamy gland, or, if present, only as a component in higher Gleason patterns as foamy macrophages [3]. Furthermore, there seems to be no specific factor that can be derived from the published cases so far, as some cases present with a normal range PSA, while others present with elevated levels [9,10,11,12,13,14,15]. Patient age varies from 47 to 81 years at the time of diagnosis, with most patients being in the range of mid-60s to mid-70s years of age [9,10,11,12,13,14,15]. The most often observed Gleason grade is that of 4, with most Gleason scores being 7 or 8 [9,10,12,13,14,15]. From the published cases that report patient outcomes, most state a favorable outcome without disease progression, with follow-up ranging from 12 to 20 months [9,13,14,15]. Two of the reports, however, indicate disease-associated demise six months after diagnosis and multiple metastases [10,11].

While cytoplasmic vacuolation and pyknotic nuclei are observed as a cytopathic effect in instances of previously androgen blockade, no such treatment was initiated in our case, prior to biopsy [20,21].

## 4. Conclusions

Clear cell renal type acinar adenocarcinoma of the prostate is rare, unrecognized by the WHO and probably underreported in day-to-day practice as a type of prostatic malignancy. Diagnosis of this entity is difficult as it is predominantly a diagnosis of exclusion and needs to be differentiated from the much more common clear cell renal cell carcinoma of the kidneys, which, in rare cases, can produce prostatic metastasis.

## Figures and Tables

**Table 1:** Immunohistochemical markers and their expression in renal-type clear cell carcinomas of the prostate.

Marker	Reaction	Supported by Literature	References
CD68	Negative	no, not reported in published cases	Current case
CK AE1/AE3 and other keratins such as CK7 and 34βE12	Positive	yes	Current case [10,11,12,13,14,15]
CD10	Negative, can have focal positivity	yes	[10,11,12,13,14,15]
PAX8	Negative	yes	Current case [10,11,12,13,14]
RCC	Negative	Yes	[10,12]
NKX3.1	Positive	No, not reported in published cases	Current case
AMACR	Positive	Yes	Current case [13,14]
PSA	Positive, can be low positive and rarely negative	Yes	[10,13,14]

## Data Availability

The original contributions presented in this study are included in the article. Further inquiries can be directed to the corresponding author.
